# Extracellular Vesicles under Oxidative Stress Conditions: Biological Properties and Physiological Roles

**DOI:** 10.3390/cells10071763

**Published:** 2021-07-12

**Authors:** Elisabetta Chiaradia, Brunella Tancini, Carla Emiliani, Federica Delo, Roberto Maria Pellegrino, Alessia Tognoloni, Lorena Urbanelli, Sandra Buratta

**Affiliations:** 1 Department of Veterinary Medicine, University of Perugia, Via S. Costanzo 4, 06126 Perugia, Italy; elisabetta.chiaradia@unipg.it (E.C.); alessia.tognoloni@studenti.unipg.it (A.T.); 2 Department of Chemistry, Biology and Biotechnology, University of Perugia, Via del Giochetto, 06123 Perugia, Italy; brunella.tancini@unipg.it (B.T.); carla.emiliani@unipg.it (C.E.); federica.delo@studenti.unipg.it (F.D.); roberto.pellegrino@unipg.it (R.M.P.); 3 Centro di Eccellenza sui Materiali Innovativi Nanostrutturati (CEMIN), University of Perugia, Via del Giochetto, 06123 Perugia, Italy

**Keywords:** Extracellular Vesicles, exosomes, microvesicles, oxidative stress, antioxidants, reactive oxygen species, redox signaling, oxidative stress-related diseases

## Abstract

Under physio-pathological conditions, cells release membrane-surrounded structures named Extracellular Vesicles (EVs), which convey their molecular cargo to neighboring or distant cells influencing their metabolism. Besides their involvement in the intercellular communication, EVs might represent a tool used by cells to eliminate unnecessary/toxic material. Here, we revised the literature exploring the link between EVs and redox biology. The first proof of this link derives from evidence demonstrating that EVs from healthy cells protect target cells from oxidative insults through the transfer of antioxidants. Oxidative stress conditions influence the release and the molecular cargo of EVs that, in turn, modulate the redox status of target cells. Oxidative stress-related EVs exert both beneficial or harmful effects, as they can carry antioxidants or ROS-generating enzymes and oxidized molecules. As mediators of cell-to-cell communication, EVs are also implicated in the pathophysiology of oxidative stress-related diseases. The review found evidence that numerous studies speculated on the role of EVs in redox signaling and oxidative stress-related pathologies, but few of them unraveled molecular mechanisms behind this complex link. Thus, the purpose of this review is to report and discuss this evidence, highlighting that the analysis of the molecular content of oxidative stress-released EVs (reminiscent of the redox status of originating cells), is a starting point for the use of EVs as diagnostic and therapeutic tools in oxidative stress-related diseases.

## 1. Extracellular Vesicles (General Concepts)

Extracellular Vesicles (EVs) are a heterogenous group of membranous particles, originating from different cell compartments and processes that are released outside the cell and retrieved in body fluids, as well as in cell culture media. Originally identified during reticulocyte maturation [1], they were initially thought to be cellular debris. Nowadays, they are recognized as important players in intercellular communication [2,3,4]. EVs allow the exchange of biomolecules, including nucleic acids, between releasing and surrounding cells. Therefore, they represent a remarkable source of circulating biomarkers for disease diagnosis and prognosis [5]. EVs can be classified in three main groups, according to their biogenesis, size, and release process: exosomes, originating from the endocytic pathway, microvesicles (MVs) (or ectosomes), derived from plasma membrane, and apoptotic bodies, released by cell undergoing apoptosis. These three EV main types display different average size: exosomes are the smaller vesicles (30–120 nm) [6] whereas MVs (50–1000 nm) [7] and apoptotic bodies (50–5000 nm) [8] have a larger average size. Despite the consensus on these findings, a complete landscape of EVs is still lacking. EVs heterogeneity is higher than recognized [9,10], as recently a new type of non-membranous particles termed exomeres has been described [11]. These are smaller than exosomes (>50 nm) and not delimited by a membrane bilayer. EVs clearly originating from mitochondria, named mitovesicles, have also been found to be released extracellularly [12]. In addition, gigantic EVs (1000 nm to 10,000 nm) have been discovered to be released by the plasma membrane of metastatic cancer cells. They are implicated in cell migration and have been defined as “large oncosomes” [13]. Although terms such as “exosomes”, “microvesicles” and “apoptotic bodies” are used in the literature to describe EV subtypes, there is currently no uniform definition of these EV subtypes based on size, density, shape, biochemical cargo or function. Indeed, there are no specific markers of EV subtypes, since markers once considered specific for exosomes, such as the CD9 and CD63 tetraspanins, are similarly present in all EVs [14]. Therefore, ISEV (International Society for Extracellular Vesicles) has recommended use of operational terms for EV subtypes, referring to a physical characteristic of EVs, such as size (for instance, “small EVs” with ranges <200 nm and “medium/large EVs” with ranges >200 nm), density (defined by the type of gradient, i.e., sucrose or iodixanol, and fraction density in g/mL), or biochemical composition (defined by the presence/absence of specific markers such as CD9 and/or CD63). Besides, functional definitions based on the definition of a specific condition of the releasing cell (hypoxic EVs, apoptotic bodies) are also possible [15].

The complexity of EV heterogenous mixture is further exacerbated by methodological problems related to their isolation. So far, most of the separation protocols rely on differential centrifugation (dUC). However, the size and density of the different EV subtypes are largely overlapping. For instance, small MVs budding from plasma membrane have the same size of exosomes (less than 200 nm), and similar density. Therefore, dUC does not allow to obtain a pure preparation of these types of vesicles. For this reason, the terms small EVs (sEVs), for EVs isolated at high centrifugal forces (100,000× *g*), enriched in exosomes, and large/medium EVs (l/mEVs) for EVs isolated at lower centrifugal forces (10,000× *g*) have been recommended. Other separation methods are available, but all of them suffer drawbacks and pitfalls, and none of them assure more than an enrichment on specific EV subtypes [16]. In fact, to obtain high recovery of EVs, in addition to dUC, precipitation with polymers and low molecular weight cutoff filtration have been also used. However, all these methods display a low specificity, with the usual presence of molecules of the same size and density. Moreover, dUC is also associated with a significant mechanical damage to EVs, due to particle deformation, aggregation and lysis, which alter their physiological properties when administered to living cells or organisms [15,17]. Other methods allowing for the recovery of EVs with a higher level of purity, but with a lower yield, have been also developed, such as size-exclusion chromatography (SEC), high molecular weight centrifugal filtration and tangential flow filtration [15,17], however, they all implicate a certain level of contamination by proteins present in the original matrix. To recover EVs with a higher level of purity, possibly including an extremely high enrichment of a single EV subtype, the best protocols available include a combination of more than one method, as for example filtration combined with SEC, dUC followed by density gradient separation and affinity and/or immuno-capture, based on the specific presence of macromolecules of EV surface, namely proteins, but also sugars or lipids [15,17]. These methods allow us to eliminate most of the no-vesicular components but are characterized by a low yield. The complexity of these methodological issues makes it difficult to compare different studies, as results may vary depending on the separation method used or on the presence of secretome contaminants, partially explaining contradictory results in the field. In addition, many studies currently available were carried out before the update of the minimal information for studies of EV guidelines [15], or did not consider them, and thus the suggested nomenclature operational terms for EV subtypes were not used, and some methodology details or physico-chemical features were not specified. Nevertheless, in our manuscript, all the reviewed studies were reported respecting the nomenclature authors used in their work, namely MVs, exosomes, or even microparticles.

EVs are characterized by the presence of a biochemically relevant content, which is reminiscent of the donor cell, but are clearly different from it, thus indicating that EV cargo loading is a specifically regulated process. Several proteomic studies have provided evidence that a few proteins are present in EVs of different tissue sources, thus representing EV markers. Among these proteins, tetraspanins, such as CD9, CD63 and CD81, and proteins related to EV biogenesis, such as Alix and Tsg101, are the most representative [14]. The lipid content of EVs is also different from that of the releasing cell, as EVs are enriched in cholesterol and sphingomyelin, as well as in saturated fatty acids, thus suggesting a composition resembling that of lipid rafts [18,19,20]. Moreover, EVs contain enzymes acting on membrane lipids, such as phospholipases and on lipid mediator biosynthesis, including Cox1 and Cox2, thus indicating that they may play a role as conveyors of bioactive lipids [21]. Finally, the most remarkable feature of EV cargo is related to the presence of nucleic acids. EVs contain many types of RNA, and specifically, they are enriched in long and short RNA, namely miRNA, but also contain mRNA, which was demonstrated to be effectively translated in donor cells [22]. The biogenesis of exosomes and MVs has not been fully elucidated, but current knowledge indicates that EV heterogeneity is mirrored by a multiplicity of biosynthetic pathways. Exosomes originate from the inward budding of late endosomes, whose lumen becomes full of IntraLuminal Vesicles (ILVs). For this reason, these endosomes are called MultiVesicular Bodies (MVBs). Upon MVB fusion with the plasma membrane through exocytosis, ILVs are released extracellularly, taking the name of exosomes. The inward budding of late endosome membrane is a key step for ILVs’ formation and can rely on either Endosomal sorting complex, required for transport, (ESCRT)-dependent, or ESCRT-independent mechanisms [23]. ESCRT machinery is based on four complexes (ESCRT 0, I, II, III), that together with accessory proteins (Alix, Tsg101), assemble on the endosomal membrane and select the cytoplasmic cargo to be loaded (ESCRT-0), promote membrane invagination (ESCRT-I and II) and operate membrane scission to release ILVs in the late endosome lumen (ESCRT-III). Alix plays a key role in cargo selection, not only by assisting the sequential association of ESCRT complexes from 0 to III, but also in association with the ESCRT-III complex alone. In fact, the sorting of tetraspanins in MVBs was demonstrated to require the ESCRT-III complex, that efficiently recruits proteins to endosomes with the help of lysobisphosphatidic acid, but not the involvement of other ESCRT complexes [24]. Besides, Alix also interacts with syntenin, the cytoplasmic adaptor of syndecan heparan sulphate proteoglycans, controlling the formation of exosomes [25]. The simultaneous inactivation of specific components of the four ESCRT complexes failed in suppressing exosome release, thus demonstrating the presence of additional mechanisms of exosome biogenesis, completely independent from the ESCRT machinery. A few of them have been characterized, that rely either on proteins or lipids. As for proteins, membrane microdomains enriched in tetraspanins have been shown to participate in exosome biogenesis by clustering into ordered structures [26]. As for lipids, the inhibition of neutral sphingomyelinase 2, an enzyme that generates ceramide from sphingomyelin, has been shown to reduce ceramide level and exosome secretion [27]. Enzymes modulating the level of phosphatidic and lysosphosphatidic acid, such as phospholipases, also affect the release of exosomes in several cell lines [28]. In summary, there is evidence that more than one biogenetic machinery is present at the same time in the same cell, suggesting that the suppression of exosome secretion is a complicated therapeutic target. MV biogenesis is characterized by the cargo accumulation on the cytosolic side of the plasma membrane, followed by membrane blebbing and fission. ESCRT proteins, such as Tsg101, have been reported to be involved in MV formation, as they are recruited on the plasma membrane by the adaptor protein arrestin domain-containing protein 1 [29]. Membrane blebbing requires localized changes in plasma membrane protein and lipid components to modulate membrane curvature and rigidity. Indeed, another sphingomyelinase, the plasma membrane-associated acid sphingomyelinase, acts locally on sphingomyelin to produce ceramide, which triggers the shedding of EVs from glial cells [30]. Several small GTPases, such as ARF6 and members of the Rab or Rho families are also implicated in the contraction of actin beneath the plasma membrane [31], an important event for MV fission. 

The biogenesis and release of EVs has been demonstrated to be increased in different stress situations. These include hypoxia [32] and conditions such as senescence [33,34] or oncogene activation [35], which are known to affect the activation status of p53 [36]. The lysosomal status was also suggested to have an impact on the release of EVs, as well as oxidative stress [37].

## 2. Oxidative Stress (General Concepts)

**Figure 1:**
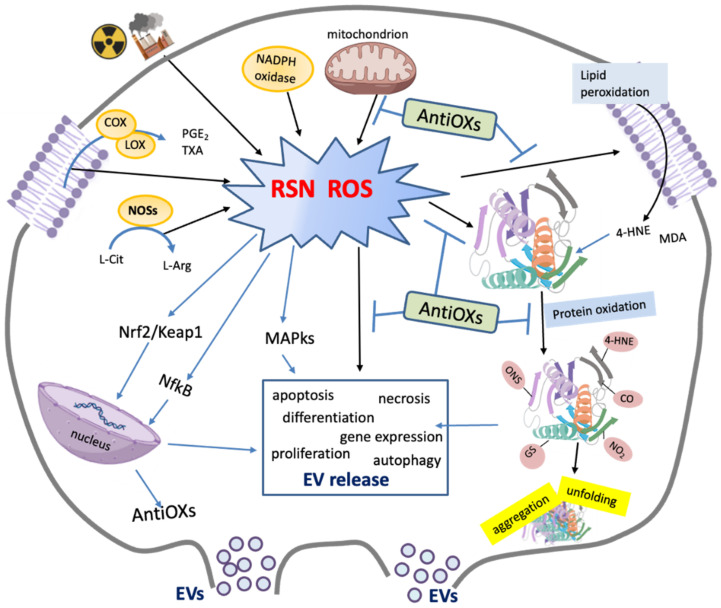
Schematic representation of main sources and effects of Reactive Oxygen Species (ROS) and Reactive Nitrogen Species (RNS). ROS and RNS are mainly generated by external stimuli (e.g., ionizing radiation and pollution), mitochondrial dysfunctions, NADPH oxidase activation, nitric oxide synthase (NOS), as well as by arachidonic acid (AA) cascade leading to the production of eicosanoids (e.g., prostaglandins (PGs), thromboxane A2 (TXA2)) by cyclo-oxygenase (COX) and lipoxygenase (LOX). Antioxidants (AntiOXs) can reduce the levels of ROS and RSN, repair or remove damaged molecules such as products of lipid peroxidation (i.e., 4-hydroxynonenal (4-HNE) and malondialdehyde (MDA)) as well as oxidated proteins (i.e., carbonylated (-CO), nytrosilated (-SNO), nitrated (-NO2), glutathionylated (-SG) proteins and 4-HNE adducts). ROS and some redox-modified proteins are involved in the signaling pathways (MAP-kinases and trascription factors (Nrf2, NfκB)) related to the modulation of cellular processes including EV release, antioxidant response and others (e.g., gene expression, cell proliferation, differentiation, migration, apoptosis, etc.).

The aerobic metabolism is linked to the production of reactive oxygen species (ROS) and reactive nitrogen species (RNS). These are radical and non-radical derivatives of oxygen and nitrogen playing important roles in physio- and pathological processes. The term ROS refers to superoxide anion radical (O_2_^•−^) hydroxyl radical (^•^OH), hydrogen peroxide (H_2_O_2_) and singlet oxygen (^1^O_2_). They are mainly generated in the mitochondria, due to their role in the oxidative metabolism (Figure 1). Other sources of ROS include oxidation of catecholamines, activation of the arachidonic acid cascade, and respiratory burst. ROS can also be produced by external stimuli such as ionizing radiation (IR), pollution, drugs or xenobiotics as well as during the Haber–Weiss and Fenton reactions [38], which are both catalyzed by iron and copper ions. The RNS are nitric oxide (NO^•^), nitrogen dioxide (NO_2_), dinitrogen trioxide (N_2_O_3_), peroxynitrite (ONOO^−^), nitrite (NO_2_^−^), nitrate (NO_3_^−^) and nitroxyl (HNO). NO^•^, which is generated from arginine by different isoforms of nitric oxide synthase enzyme (NOSs), is able to rapidly diffuse across lipid bilayers (Figure 1). Its reaction with ROS leads to RNS such as ONOO^−^/peroxynitrous acid (ONOOH). The cross-talks between ROS and RNS, as well as between their sources make often difficult to establish what is the species which plays a specific role in physiological or pathological conditions [39].

Being highly reactive, ROS and RNS can induce oxidative modifications of carbohydrates, lipids, proteins, and DNA, with dangerous consequences for cell integrity and function. However, the aerobic cell has learned to counteract the production and harmful effects of ROS developing antioxidant defenses including enzymes (i.e., superoxide dismutase (SOD), catalase (CAT), glutathione peroxidase (GPX), glutathione reductase (GSR), peroxiredoxin (PRDX) and thioredoxin (TRX)) and bioactive substances (i.e., GSH, vitamins A and E). The members of the antioxidant machinery modulate the redox status of the cell by scavenging radical species and repairing (or removing) oxidatively damaged molecules (Figure 1).

Overall, the cellular homeostasis depends on balance between pro- and antioxidants. Physiological levels of ROS and RNS are needed to normal cell functioning, as they are involved in intra- and intercellular signaling [39,40,41] (Figure 1). However, when ROS levels exceed antioxidant defenses, “oxidative stress” occurs [42,43,44]. It is a harmful condition underlying the onset of a variety of pathologies such as cancer, diabetes, Alzheimer disease, and parkinsonism [45,46,47,48].

Overall, cells utilize the low in vivo concentrations of ROS and RNS to regulate metabolic processes, gene expression, cell cycle progression, cytoskeletal organization, antigen processing, cell proliferation, differentiation, migration, and apoptosis and EV release [37,48] (Figure 1). This emerging finding stimulated, in the last decades, the redefinition of the concept of “oxidative stress” in “an imbalance between oxidants and antioxidants in favor of the oxidants, leading to a disruption of redox signaling and control and/or molecular damage” [43,44]. However, it is also crucial to consider that the word ‘stress’ refers to a ‘general adaptation syndrome’ according to Selye’s definition [49], that implies a cellular and body response to return to redox initial conditions, minimizing the effects [50]. Indeed, pro-oxidant conditions are able to stimulate the expression of antioxidant enzymes activating transcription factors such as nuclear factor erythroid 2-related factor 2 (Nrf2)/Kelch-like ECH-associated protein 1 (KEAP1), Nf-κB, AP-1, MAP-kinases, NPR1/TGA, which are involved in key adaptation processes [39].

The extent of oxidative damage on molecular target depends on various factors including their location and concentrations, the possibility to generate secondary damaging events (e.g., chain reaction) and the ability to reacts with antioxidants or scavengers [51].

Polyunsaturated fatty acids (PUFAs) of membrane phospholipids are the most ROS-sensitive lipids. Briefly, prooxidants abstracting and allylic hydrogen atom initiates an autocatalytic chain reaction that may lead to the formation of lipid-peroxyl radical (ROO^•^), lipid peroxide (ROOH), as well as reactive aldehydes such as 4-hydroxynonenal (4-HNE) and malondialdehyde (MDA) [52,53]. Lipid peroxidation induces alteration of the membrane physical properties, with consequences on phospholipid dynamism, membrane shedding, membrane fluidity and permeability [54]. Moreover, lipid peroxidation can be the result of the controlled peroxidation of PUFAs by the action of enzymes such as lipoxygenase and cyclooxygenases [53] that produce lipids mediators (i.e., prostaglandins, leukotrienes and thromboxanes) involved in inflammation and immune response. The reactive aldehydes 4-HNE and MDA have a long lifetime and can move intracellularly or extracellularly. 4-HNE can form covalent adducts with proteins leading structural changes (i.e., increased β-sheet conformation) with consequences in protein turnover and proteasome activity [54,55]. Moreover, 4-HNE has been described as a “second messenger” in various cellular signaling pathways modulating cell proliferation, differentiation, apoptosis and autophagy [56,57,58,59,60,61]. Recently, accumulation of lipid-based reactive oxygen species has been associated to ferroptosis, a form of iron-dependent regulated cell death that is characterized by an increase in intracellular redox-active iron, oxidation of phospholipid PUFA and loss of lipid peroxide repair capacity [62,63].

Proteins are the most abundant components of cells and biological systems, and the major target of ROS and RNS [51,64]. Amino acids can react directly with both ROS and RNS leading to the formation of various oxidative and nitrosative species, or indirectly with reactive products generated by oxidation of other molecules (i.e., lipids), forming adducts. The resulting wide variety of oxidative products may induce irreversible or reversible modifications of protein structure and folding, altering their function. Nevertheless, the redox signaling is mainly linked to the oxidative modification of amino acid side chains. The redox-driven post-translational modifications allow reversible activation/deactivation of protein targets similarly to those obtained by phosphorylation and acetylation [44]. The redox modification of histone (nitrosylation, carbonylation, or glutathionylation) is, for example, a crucial tool of epigenetic regulation [65] and gene expression modulation, whereas redox regulation of protein kinases modulates apoptosis or growth signaling [66,67]. The amino acids particularly susceptible to oxidation are the sulfur-containing amino acids cysteine (Cys) and methionine (Met), followed by proline, histidine, tryptophan, selenocysteine and selenomethionine. The concentration of reactive species can be crucial. H_2_O_2_, at nM range, mediates reversible oxidation of Cys residues within proteins, from thiolate anion (Cys-S-) to sulfenic form (Cys-SOH), inducing allosteric changes and protein function modifications, whereas higher levels of H_2_O_2_ oxidize thiolate anions to sulfinic (SO_2_H) or sulfonic (SO_3_H) species, which can result in irreversible alterations and permanent protein damage.

The most harmful, irreversible, oxidative protein modification is protein carbonylation, broadly used as a biomarker of oxidative protein damage and oxidative stress [68,69]. It consists in the formation of reactive aldehyde or ketone residues on proteins that can be generated through different mechanisms (e.g., oxidative cleavage of the peptide bond and by a direct attack of ROS and RNS on the side chains of lysine (Lys), arginine, proline, threonine, and histidine residues etc.) [68,69]. Carbonylation has been interpreted as a signal for damaged protein degradation, as carbonylated proteins are more susceptible to proteolytic degradation than unmodified proteins or those with different oxidative modifications [70,71]. The accumulation of reactive carbonylated species has been detected in various disorders, including metabolic diseases, hematological malignancies, lung and kidney diseases, sepsis, amyotrophic lateral sclerosis, cystic fibrosis, and oxidative stress-related neurodegenerative diseases [48,72,73,74,75]. Redox proteomics evidenced, in different tissues, a large number of carbonylated protein species that can affect cellular pathways such as the proteasomal system in different pathological conditions [75,76,77]. A different site-specific carbonylation susceptibility across amino acids, and among distinct proteins, has been also demonstrated [78,79].

Another oxidative modification of proteins is tyrosine nitration, which is mainly generated through the oxidation of tyrosine by peroxynitrite and peroxynitrite-derived radicals or by the myeloperoxidase activity. Tyrosine nitration is highly selective (relatively few specific proteins are preferential targets) [80] and can cause dramatic changes in protein structure, inducing loss or gain of function as well as impairment of cellular processes relying on tyrosine phosphorylation cascades or protein turnover [81,82]. Many studies have suggested specific roles of 3-nitro-tyrosine in the functional changes related to disease conditions [82,83,84]. Moreover, tyrosine nitration can induce autoimmune responses stimulating the production of specific antibodies against nitrated proteins [83].

Among reversible modifications, Cys-S-nitrosylation and S-glutathionylation, as well as Met oxidation to Met sulfoxide, play a crucial role in the regulation of protein function and metabolic processes. The oxidation of Met results in the formation of two diastereomeric forms of Met sulfoxide (S-MetO or R-MetO), that can be reduced back to Met by Met sulfoxide reductases, MSRA and MSRB, respectively. Impairment of these enzymes can lead to insufficient repair of oxidized MetO causing protein structure alterations, protein aggregation and/or loss of functions underlying the onset and progression of diseases including neurodegenerative disorders and cancer [85,86,87]. Met oxidation makes Met more hydrophilic, causing protein structural alterations and increasing vulnerability of amino acids to carbonylation [88]. S-nitrosylation results from addition of nitrosyl group to thiol of specific Cys residues of proteins. It is stimulated by high-pro-oxidant condition and low level of antioxidants, whereas it is reduced by NOS inhibitors. Protein S-nitrosylation levels can also depend on denitrosylation reactions consisting in enzymatic or non-enzymatic cleavage of S-nitrosyl group from Cys [89]. Although the intracellular S-nitrosylation protein level is relatively low, it has been linked to pathological conditions in different ways [90,91,92]. S-nitrosylation can regulate at least 3000 proteins involved in different biological processes. Hess and Stamler described S-nitrosylation as pleiotropic regulator of other PTMs such as phosphorylation, acetylation and ubiquitination [93]. Nrf2, p53, Nf-κB and hypoxia-inducible factor-1α (HIF-1α) are examples of transcription factors that can be regulated by S-nitrosylation. Proteins involved in apoptosis such as VDAC, Bcl-2 and some caspases are also regulated by S-nitrosylation [89,94]. For example, the constitutive nitrosylation of caspase-3 is modulated by mitochondrial Trx/Trx reductase system (Trx2/TrxR2), that regulates death receptor-mediated apoptosis in lymphocytes, melanoma and hippocampus cells [95,96]. Low molecular weight thiols, such as GSH and coenzyme A (CoA) can both be nitrosylated, generating S-nitrosoglutathione and S-nitroso-coenzyme A, respectively, that are in thermodynamic equilibria with Cys-nitrosylated proteins [97].

GSH can also bind specific protein by disulfide linkages with Cys residues [98]. This process, defined as protein S-glutathionylation can be either spontaneous or enzymatically driven, depending on the redox state of intracellular glutathione pools [99,100]. In particular, glutathionylation of proteins is catalyzed by glutathione S-transferases and can protect or mitigate protein oxidation. Protein S-glutathionylation is also a tool to modulate protein function and cellular processes including apoptosis, calcium homeostasis [101], glycolysis [102] and cytoskeleton dynamism [103]. For example, glutathionylation of p53 can induce accumulation of damaged mitochondria and apoptosis [104].

Protein thiols, which represent the main cellular redox buffer, beyond doubt play a central role in redox signaling [105]. Their functions depend on their redox status which is under the control of thiol-containing systems including GSH and TRX. The TRX system, which is composed by NADPH, TrxR, and TRX, regulates protein dithiol/disulfide balance through its disulfide reductase activity, modulating the activity of many redox-sensitive transcription factors. The cytosolic and mitochondrial TRX systems, together with the GSH-glutaredoxin system, modulate the cellular redox environment [106]. GSH that under oxidative stress is converted by GPXs into GSSG to reduce targets, carries out several organelle-specific functions. The ratio GSH/GSSG is known as a redox state regulator and indicator [107]. GSH is predominantly present as a reduced tripeptide into cytosol, nucleus, and mitochondrion, where it is crucial to proper protein folding and activity, whereas high GSSG levels in ER seems to support the formation of disulfide bonds and the functional conformation of nascent peptides, especially of secretory and membrane proteins [107].

GSH and TRX systems are also involved in the modulation of Nrf2 signaling that represents the major mechanism of adaptative response to oxidative stress [108]. Nrf2 is a transcription factor that interacts with Keap1 in the inactive form. High levels of ROS induce oxidation of redox-sensitive cysteine residues of Keap1, resulting in dissociation of Keap1 from Nrf2 [109]. Subsequently, Keap1 can be ubiquitinated and degraded by proteasomal systems, while Nrf2 translocates into the nucleus, forming a heterodimer with the small MAF protein and binding the antioxidant-responsive elements (ARE) on DNA. These control the expression of over 250 genes encoding for enzymes and proteins involved in redox homeostasis and antioxidant response [110], such as enzymes acting in ROS catabolism (SOD, GPX, PRDX), in oxidized factors regeneration (GSR, TrxR), in GSH synthesis (glutamate-cysteine ligase), in stress response (heme oxygenase) as well as enzymes involved in carbohydrate and lipid metabolism, autophagy, apoptosis and DNA repair [108,111]. Growing evidence has demonstrated that Nrf2 can be also regulated trough phosphorylation by protein kinases [112] or acetylation by histone acetyltransferases (HATs) [111].

Moreover, it has been reported that Nrf2 can be regulated by circulating microRNAs (miRNAs) included into EVs [110]. Exosomal-Nrf2 and exosomal-Nrf2-mediated products can also activate antioxidant signaling pathways and regulate oxidative homeostasis in different target cells [113,114]. However, as it is reported in the next section, the release and the uptake of EVs can be differently related to oxidative stress with various implications.

## 3. Biochemical and Signaling Features of EVs Released under Oxidative Stress Conditions

**Figure 2:**
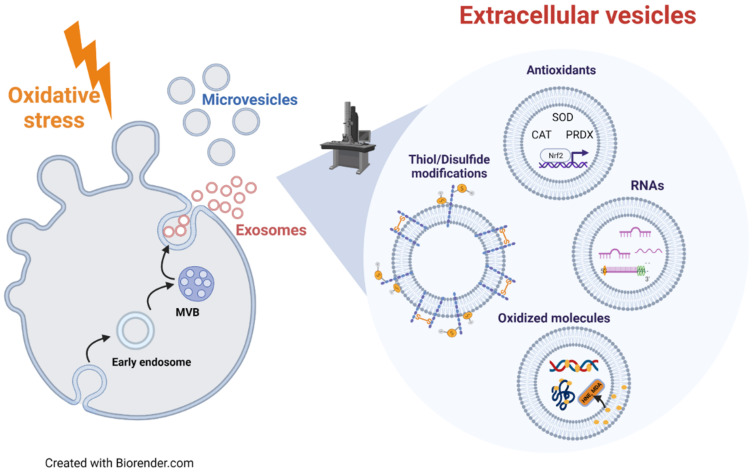
Link between EVs and redox status of originating cells. Oxidative stress conditions influence the release and the molecular cargo of EVs, both exosomes and microvesicles, which can modulate the redox status of target cells. Oxidative stress-related EVs can carry components of antioxidant machinery (SOD, CAT, PRDX and Nrf2), oxidized molecules, specific RNAs involved in cell response to oxidative stress and an altered content of protein thiol/disulfide groups. The delivery of these molecules via EVs could be a way used by oxidatively stressed cells to eliminate damaged/toxic molecules. In addition, the oxidative stress-related molecular cargo of EVs can be transferred to target cells, thus modulating their response to oxidative stress.

Cells release EVs to spread signaling molecules, respond to stress stimuli, and to remove waste or unneeded material. Oxidative stress is a complex cellular condition regulating the level and the content of released EVs. An excess of ROS can affect cell signaling, altering the amount and molecular cargo of EVs (Figure 2). EVs can convey oxidized lipids and proteins, and this cargo can be responsible for EV-mediated detrimental effects on target cells [37,115] (Figure 2). In agreement with this finding, recently, much evidence has indicated a critical role of EVs in the development and progression of oxidative stress-related pathologies, i.e., cancer (including metastasis), neurodegenerative and inflammatory diseases [37,116,117]. Conversely, the cargo of EVs released under oxidative stress also includes antioxidant molecules that modulate the oxidative stress response in target cells, protecting them against further injury [37,115] (Figure 2). Therefore, the molecular cargo of oxidative stress-related EVs represents a useful marker of the redox status of cell/tissue of origin, and a useful tool to define the role of EVs in the progression of oxidative stress-related diseases. The knowledge of the molecular mechanisms modulating EV release is crucial for the development of therapeutic strategies for oxidative stress-related pathologies, based on either the removal of harmful molecules or the shuttling of antioxidants via EVs.

### 3.1. Modulation of EVs Release by Oxidative Stress Conditions

The first issue taken into account is the well demonstrated increase of EV release under oxidative stress conditions. Indeed, numerous studies carried out in vitro on different cell lines demonstrated that different pro-oxidant stimuli increase the amount of released EVs, including either MVs or exosomes. As an example, Atienzar-Aroca et al. [118] showed that oxidative stress conditions induced by ethanol exposure prompted the release of exosomes from retinal pigment epithelium cells. Similarly, human retinal pigment epithelial RPE-1 cells treated with the ROS-inducer doxorubicin released a greater amount of EVs, compared with non-treated cells [119], and oxidative stress-induced by calcium ionophore A23187 stimulated the shedding of EVs in HEK293 [120]. Furthermore, van Meteren et al. [121] demonstrated that polycyclic aromatic hydrocarbons (PAHs), which are environmental contaminants, induce the release of EVs, mainly exosomes, by primary rat hepatocytes and the WIF-B9 cell line. The link between oxidative stress and release of EVs was confirmed by the evidence that PAH-induced EV release was counteracted by co-incubation with antioxidants (i.e., thiourea or vitamin E) [122]. The effect of oxidative stress on EV release seems to depend on cell type and stimulus. For instance, cell exposure to 4-HNE enhances the release of Tissue Factor-positive EVs by endothelial cells and fibroblasts, but not by monocytes [123]. Cigarette smoke extracts (CSE) and acrolein induced the release of CD63^+^/CD81^+^ EVs by human lung epithelial BEAS-2B cells, whereas H_2_O_2_ did not show any effect. Coherently, antioxidants (i.e., NAC or GSH) reduced the release of CSE-EVs [124].

Several studies focused on the effect of cell redox status on EV biogenesis mechanisms. Yarana reports in a recent review [37] the different EV biogenesis mechanisms that are known or expected to be influenced by redox status of originating cells, analyzing current knowledge on the effects of the chemotherapeutic drug doxorubicin, an oxidative stress inducer, on EV release. Doxorubicin activates p53 [125], which further upregulates the tumor suppression-activated pathway 6 (TSAP6), an endosomal membrane protein implicated in MVB formation [126]. It induces mitochondrial impairment by acting on the electron transport chain. The consequent mitochondrial membrane potential loss inhibits the influx of Ca^2+^, which results in an elevation of cytosolic [Ca^2+^], a key event that induces both MV and exosome release by promoting membrane blebbing as well as fusion of MVBs with plasma membrane [127]. Doxorubicin induces an impairment of autophagy [128,129], a cellular event closely related to exosome biogenesis [130]. In fact, conditions that promote autophagy drive MVBs toward lysosomes rather than the plasma membrane, thus inhibiting exosome release [131], whereas the inhibition of autophagic trafficking promotes the release of exosomes [132,133]. Doxorubicin blocks the autophagic flux and promotes accumulation of autolysosomes by inducing an alkalinization of lysosomal lumen due to the inhibition of V-ATPase [134]. Thus, the doxorubicin-induced EV release bypasses the block of the autophagic flux and reduces the cellular overloading of damaged/oxidized molecules. In agreement, drugs able to alkalinize lysosomal pH, i.e., bafilomycin A or chloroquine, induce the release of EVs [135,136].

Lipid membrane remodeling seems to be one of the key events responsible for the increased release of EVs from PAHs-treated hepatocytes [121]. PAH treatment causes a decrease in total cellular cholesterol content and an increase of membrane fluidity, an event that could promote EV release. In addition, EVs released by PAH-treated hepatocytes present higher cholesterol content and higher levels of ESCRT machinery proteins (Tsg101 and Alix), compared with EVs from untreated cells. These results are in line with the demonstration that ESCRT complex forms clusters inducing ordered membrane microdomains in a cholesterol-dependent manner, in artificial membranes [137]. Cholesterol levels were reported to be a regulator of EV release in several cell lines [138], as it is essential for the movement of MVBs toward plasma membranes along microtubules [139] and for membrane fusion processes [140].

Other membrane rearrangements that modulate membrane fusion and EV release are changes in the level of cell surface protein thiol groups [115,124,141]. Membrane impermeable thiol blocking compounds, i.e., DTNB or bacitracin, trigger exosome release in BEAS-2B cells. Similarly, the exosome release induced by CSE and acrolein is accompanied by the depletion of cell surface thiols [124]. Szabo-Taylor et al. [141], demonstrated a role for EVs in the modulation of cell surface thiol levels, an event occurring during immune cell activation. The exposure of monocytes to inflammatory stimuli (LPS or TNF) induces the release of EVs characterized by low levels of exofacial thiols, and this event seems to contribute to the increased levels of thiol groups on the cell surface. Thus, cells might rapidly enhance their plasma membrane thiol levels by shedding oxidized (less reduced) plasma membrane patches as EVs [141]. These authors also demonstrated that the antioxidant enzyme PRDX 1 is secreted via EVs in the oxidated form associated to the cell’s surface. PRDXs contain cysteine residues that need to be oxidized prior to their inclusion in exosomes. As soluble PRDX 1 was shown to induce an inflammatory response in monocyte cells [142], it is possible that the secretion of the oxidized, inactive PRDX 1 via EVs represent a route used by cells to eliminate this protein in a safe, non-harmful form [141].

### 3.2. Modulation of EV Cargo by Oxidative Stress Conditions

EVs released under oxidative stress can mediate either protective or harmful signals in target cells exposed to the same stress, depending on their specific biochemical cargo. Thus, the knowledge of the molecular cargo of oxidative stress-released EVs could be predictive of the effects that they exert on target cells. Exosomes released by mouse mast cell line (MC/9) exposed to H_2_O_2_ can induce tolerance to oxidative stress in target cells. Indeed, the pre-treatment with exosomes released under conditions of oxidative stress increases the viability of cells exposed to H_2_O_2_, compared to cells pre-treated with exosomes harvested from normal conditions. This protective role seems to be partly mediated by the mRNA exosomal cargo [143]. In fact, microarray analysis shows that mRNA content of exosomes from H_2_O_2_-treated cells was remarkably different from that of exosomes produced by cells cultured under normal conditions, whereas no differences were observed in the levels of carbonylated proteins [143].

Interesting information regarding the biochemical composition of oxidative stress-related EVs derives from studies on airway epithelial cells exposed to CSE or cigarette smoke condensate (CSC). One of these studies examined the content of small noncoding RNAs (sncRNAs) in EVs released by human small airway epithelial (SAE) cells treated with CSC [144]. Significant changes were observed in the content of microRNA (miRNAs) and Piwi-interacting RNA (piRNAs). NGS analysis identified 289 miRNAs in EVs, with 5 significantly upregulated and 3 downregulated in CSC-EVs, compared to controls. A total of 62 piRNAs being detected in SAE-derived EVs, with 5 significantly downregulated and 2 upregulated in CSC-EVs. Real-time PCR confirmed that CSC treatment led to the upregulation in EVs of novel miRNAs, namely miR-3913-5p, miR-574-5p, and miR-500a-5p, whose potential biological targets seem to be involved in lipid transport/binding and gene transcription regulation [144].

Proteomic analysis of EVs released by airway epithelial cells exposed to CSE confirmed the reliability to predict EV biological effects on the basis of their protein composition [145]. EVs released by BEAS-2B cells treated or not with CSE demonstrate that 33% of the proteins are differentially expressed between the two conditions. Functional enrichment analysis for GO domain “cellular component” reveals that “plasma membrane” and “cell surface” proteins are upregulated in CSE-EVs. Concerning the “molecular function”, CSE-EVs are enriched in proteins with “GTPase activity” and “receptor activity”. Functional enrichment for “biological process” and “biological pathway” reveals an upregulation of proteins related to immunity (“immune response”, “adaptive immune system”) and communication (“cell communication”, “signal transduction”) in CSE-EVs. The upregulation of proteins involved in “haemostasis” and “platelet activation, signaling and aggregation” indicated that CSE-EVs convey pro-coagulant signals [145]. This hypothesis was confirmed by in-vitro experiments demonstrating that CSE-EVs exert pro-coagulant effects, which depend on the high levels of Tissue Factor and phosphatidylserine present on CSE-EVs [145].

Biasutto et al. [146] analyzed the protein and phosphoprotein composition of exosomes released by human retinal pigment epithelial ARPE-19 cells incubated with methyl viologen or rotenone. The identification of proteins in their phosphorylated state was important because it might reflect the activation of specific signaling pathways in originating cells. Exosomes released from cells under normal conditions revealed 72 proteins and, among them, 41 were specifically detected in their phosphorylated or cleaved form. When compared to exosomes released under oxidative stress conditions, changes in the levels of phosphorylation were observed in proteins belonging to pathways regulating cell proliferation, survival, and energy metabolism. Oxidatively stressed cells and exosomes presented a downregulation of proteins related to pro-survival pathways and an upregulation of pro-apoptotic proteins [146]. Thus, oxidatively stressed cells might transfer via EVs molecules related to signaling pathways regulating cell metabolism and death.

In addition to RNA and proteins, EVs released under oxidative stress conditions convey oxidized lipids generated from peroxidation of cell membrane phospholipids. Lipid peroxidation products disseminated to other cells via EVs seems to mediate relevant biological effects. Endothelial cells exposed to peroxides release MVs containing oxidized phospholipids able to activate neutrophils and monocytes [147,148]. Consistently, EVs released by HEK293 or HUVECs under oxidative stress or synthetic EVs subjected to limited oxidation carry oxidized hydro(pero)xylated phospholipids, such as hydroperoxy eicosatetraenoic-phosphatidylethanolamines ([H(p)ETE-PEs]), that are able to activate Toll-like receptor 4, similarly to the microbial signal LPS [120]. Interestingly, oxidatively stressed EVs induce a transcriptional response in bone marrow-derived macrophages that differ in some respects from that induced by LPS, as oxidatively stressed EVs also induce the expression of several genes whose products contribute to the resolution of inflammation [120].

Another important issue is the capability of EVs to convey antioxidants, thus acting as ROS scavengers, as well as enzymes involved in ROS production [149]. The capability of EVs to act as scavenger or producer of ROS seems to be affected by the physio-pathological conditions and redox status of the releasing cells. In this context, it must be considered that EVs produced by stem cells have the ability to reduce ROS levels in target cells. Exosomes derived from human umbilical cord mesenchymal stem cells (MSCs) exert antioxidant and antiapoptotic effects in cisplatin-induced acute kidney injury both in vivo and in vitro [150]. Menstrual stem cell-derived exosomes reduce the ROS production and the secretion of pro-angiogenic factors in prostate cancer PC3 cells [151]. Besides, both healthy or oxidatively stressed cells release EVs containing enzymes (i.e., catalase, SOD, peroxidases, reductases, PRDX, TXN) and molecules (i.e., glutathione) with antioxidant activity [37,149]. As an example, MVs containing SOD2 and catalase produced by stimulated T-lymphocytes exert protecting effects on Actinomycin D-induced apoptosis in HUVEC, by reducing ROS levels and inducing SOD1 expression [152]. Padel et al. [153] demonstrated that pancreatic cancer cells treated with chemotherapeutic gemcitabine release exosomes enriched in ROS-detoxifying genes (SOD2 and catalase), which are transferred to neighbor cells protecting them from gemcitabine-induce oxidative stress. Exosomes derived from bone marrow MSCs treated with H_2_O_2_ protect C-kit^+^ cardiac stem cells against oxidative stress-induced cell death by transferring miR-21 [154]. Furthermore, exosomes from granulose cells treated with H_2_O_2_ present high levels of Nrf2 and its downstream antioxidants (catalase, PRDX1 and TXN1) mRNAs, which once internalized by target cells counteract oxidative stress [113]. As previously reported, macrophages stimulated by LPS released EVs containing the antioxidants enzyme PRDX in its oxidized/inactive form [155]. Exosomes derived from human induced pluripotent stem cells reduce ROS production and senescence features in aged MSCs through the transfer of their associated PRDXs [156]. Altogether, these results demonstrate that oxidatively stressed cells release EVs that can contain an antioxidant cargo, which can be horizontally transferred to target cells protecting them by oxidative stress damages.

Conversely, EVs can autonomously produce ROS, both in extracellular fluids, or in target cells, after their internalization [149]. Indeed, the existence of NADPH oxidase (NOX), an enzyme that synthesized ROS, has been demonstrated in both MVs [157] and exosomes [158]. Exosomes from bone marrow-derived macrophages are able to transfer their associated NOX2 to injured axons, thus inducing an increase of ROS levels. This event triggers the activation of PI3K–Akt signaling pathway and the consequent regenerative outgrowth [158].

## 4. EV Role in Oxidative Stress-Related Diseases

In the previous section, we reported evidence derived from in-vitro studies performed in different cell lines, where oxidative stress was induced by different oxidants/stressors. To have a more reliable picture regarding the role of EVs in redox-mediated intercellular signaling, in this section we report evidence regarding the role of EVs in the pathogenesis and progression of oxidative stress-related diseases.

**Figure 3:**
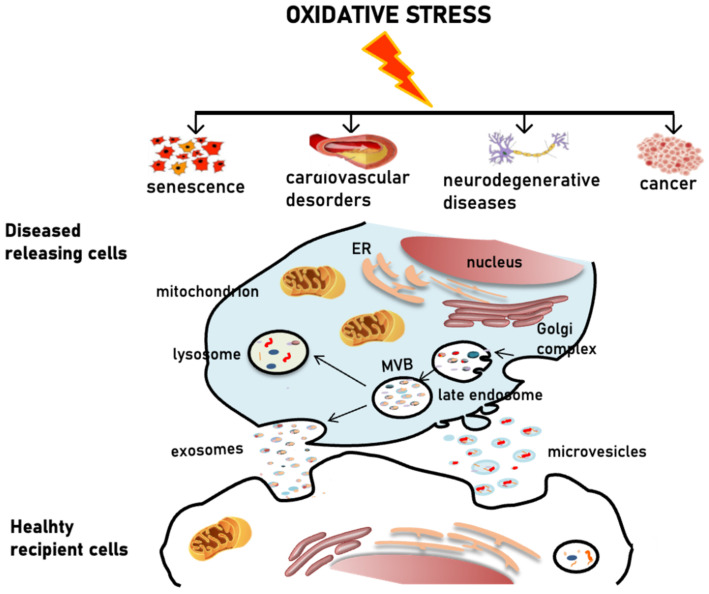
Schematic representation of the EV-mediated pathophysiology of oxidative stress-related diseases. Oxidative stress is associated with the pathogenesis of several disorders. Pro-oxidant conditions induce the release of EVs. Diseased cells can eliminate ROS and oxidized toxic molecules via EVs, thus spreading pathological alterations by delivering their cargo to healthy cells.

Oxidative stress is known to be associated with various pathologies [159]. Aging and age-related diseases such as cardiovascular complications, neurodegenerative disorders and cancer, are all characterized by oxidative stress conditions which result from the accumulation of free radicals, due to metabolic alterations, reduced clearance of damaged proteins and organelles, progressive decrease in the efficiency of antioxidant machinery [149]. Aging and age-related diseases are also commonly associated to inflammation. Pro-oxidant and pro-inflammatory conditions have been reported to induce EVs release [115] and, consistent with this, senescence, as well as cancer, cardiovascular and neurodegenerative diseases, are all associated with an increased production of EVs characterized by an altered biochemical composition [35,160,161,162,163] (Figure 3). EVs, especially exosomes, are strongly implicated in the pathophysiology of diseases associated to oxidative stress conditions (Figure 3). As reported in the previous section, ROS and oxidized molecules can be sorted into EVs and delivered to neighboring cells spreading damage to the tissue and triggering inflammation [116] (Figure 3). On the other hand, EVs released under oxidative stress conditions can protect cells from the same insult by transferring antioxidant enzymes acting as ROS scavengers [116].

Evidence points to the involvement of EVs in intercellular communications in senescence and aging [164]. The induction of cellular senescence is considered a hallmark of aging and is also a driving factor of some age-related disorders such as cancer, atherosclerosis and Alzheimer’s disease. Cellular senescence is characterized by a stable cell cycle arrest and the senescence-associated secretory phenotype (SASP), a proactive secretome able to influence tissue homeostasis by means of intercellular communication. Although the classical SASP is characterized by soluble factors, growth factors, and matrix remodeling enzymes, EVs are now emerging as novel SASP components [34]. EV-SASP cargo includes interferon, ephrin-related and antioxidant proteins, miRNAs and DNA fragments. Moreover, metabolites and lipid mediators that induce an oxidative microenvironment have also been found [164]. Interestingly, Borghesan et al. showed that EV-SASP can induce paracrine senescence in healthy cells by mediating a delay in proliferation and inducing an increase in the expression levels of diverse biomarkers of senescence [165]. In particular, authors demonstrated that the interferon-induced transmembrane protein 3, IFITM3, is partially responsible for this phenotype. Besides, it has been reported that small EVs isolated from primary fibroblasts of young human donors ameliorate some features of aging in old fibroblasts and a variety of tissues in old mice [166]. These EVs have glutathione S-transferase intrinsic activity. They decrease oxidative stress and lipid peroxidation, thus ameliorating senescence and aging both in vitro and in vivo. Authors suggest a potential use of small EVs in regenerative therapy in aging. Altogether, these studies highlight the importance of EVs as intercellular communication mediators in cellular senescence and aging.

As recently reviewed by Kahroba et al., exosomes containing Nrf2 and Nrf2-induced products can be transferred to recipient cells and activate anti-stress defense mechanisms in recipient cells [114]. Nrf2, as above described, is a redox-sensitive transcription factor actively involved in many physiological and pathological conditions, such as inflammation and age-related diseases, able to modulate both anti-inflammatory and antioxidant responses. Once activated, Nrf2 induces the expression of antioxidants, anti-inflammatory, cytoprotective, and detoxification genes and can transmit its effects to other cells trough exosomes. Exosomes released by human embryonic stem cell promote the restoration of antioxidant response and induce anti-aging processes in vascular endothelial cells trough the activation of Nrf2 signaling pathway [167]. The induction of Nrf2 signaling pathway mediated by exosomes released from ox-LDL treated-human umbilical vascular endothelial cells exert an anti-atherosclerosis effect by suppressing dendritic cell maturation [168]. In the brain, the Nrf2-ARE axis regulates the activation of detoxifying and antioxidant enzymes in astrocytes. These exert a neuronal protective role by secreting exosomes containing antioxidant molecules in response to oxidative injuries. These exosomes are delivered to neurons and preserve them from ferroptosis-like cell death [169]. Conversely, EVs seem to be implicated in the Nrf2 dysregulation observed in heart failure. Nrf2 plays a critical role in controlling redox homeostasis in cardiomyocytes and heart failure state is associated with oxidative stress in cardiac cells, and concomitant reduced Nrf2 protein expression [149]. Consistent with this, it has been reported that, in stress conditions, different types of cardiac cells can secrete EVs enriched with various Nrf2-targeting miRNAs that, once internalized by neighboring cells, contribute to repress the Nfr2 signaling pathway spreading the pathological alterations [110].

Further evidence indicates that EVs may be mediators of oxidative stress and inflammation in cardiovascular system, thus contributing to cardiovascular diseases and atherosclerosis. Circulating EVs have been shown to increase significantly in cardiovascular diseases and EVs released by leukocytes, erythrocytes, smooth muscle and endothelial cells strongly accumulate in atherosclerotic plaques, favoring local inflammation [170,171]. Moreover, endothelial microparticles have been reported to reduce the endothelial NO bioavailability, thereby impairing the protective role of blood vessels endothelial lining, and contributing to the development and progression of atherosclerosis [171], and it has been reported that heparin-binding EGF-like growth factor-positive microparticles derived from endothelial cells stimulate ROS production and pro-inflammatory responses in endothelial cells, through the direct interaction with the EGFR receptor [172]. These studies support the potential role of EVs in cardiovascular diseases through the induction of oxidative stress and inflammation and the promotion of progressive endothelial cell dysfunction.

Oxidative stress also plays a critical role in the pathogenesis of neurodegenerative disorders, as many studies report the presence of high levels of reactive oxidants and an impaired antioxidant defense response in the brain of patients affected by neurodegenerative diseases. Depending on the specific neurodegenerative disease, oxidative stress may be the consequence of a number of factors such as pathological protein aggregation, mitochondrial dysfunction, deregulated antioxidant pathways, specific neurotransmitter metabolism or inflammation [173]. Exosomes are secreted by several brain cells including neurons, astrocytes and microglia and it has been reported that their release is increased by oxidative stress [163,174]. Moreover, exosomes released by pathological cells have been shown to be involved in neurodegenerative processes by spreading their pathological alterations to neighboring healthy cells [175,176]. For instance, Alzheimer’s disease is characterized by both neuronal chronic inflammation and oxidative stress associated with the formation of Aβ plaques and neurofibrillary tangles that lead to irreversible neuronal dysfunction culminating in cell death [177,178]. Oxidative stress has been proposed to contribute to the Aβ production and accumulation which, in turn, induce oxidative stress thus creating a vicious circle [163,177]. Accumulated Aβ can be secreted extracellularly via exosomes and the Aβ containing-exosomes have been reported to be involved in different stages of disease pathogenesis and progression, including Aβ production, accumulation, and clearance as well as plaque formation [163]. In particular, oxidative stress induces the release of exosomes containing β- and γ-secretase contributing to the Aβ production in target cells [179,180]. This evidence indicates that exosomes can foster the propagation of Aβ protein aggregation and tau hyperphosphorylation, triggering neuroinflammation and oxidative stress and spreading the pathological alterations associated with AD [181,182]. Conversely, exosomes were also reported to exert a neuroprotective role by promoting Aβ clearance. Yuyama et al. observed that neuronal exosomes carrying Aβ on their surface in a nontoxic form, associated to glycosphingolipids, are internalized by microglia. In these cells, exosome-bound Aβ is delivered to lysosomes by the endocytic pathway and degraded [183]. The same authors also demonstrated that intracerebral infusion of cultured neuron exosomes in AD transgenic mice decreased Aβ deposition, improving the pathogenesis, and suggesting exosome supplementation or induction of exosome production as a possible therapeutic strategy for AD [184]. These contradictory results clearly underline the necessity to carry out further investigation in the field.

Exosomes also contain miRNAs and, more specifically, several miRNAs released from exosomes have been associated to neurodegenerative disorders, including AD. For instance, recent studies indicated that exosome-derived miRNAs can modulate the expression of both the amyloid precursor and tau proteins, suggesting that deregulation of exosome-associated miRNAs could be implicated in AD progression [185,186]. It is well known that oxidative stress can affect the expression of miRNAs regulating genes involved in oxidative stress responses [187]. In this regard, Li et al. [188] demonstrated that soluble Aβ peptides, which have been reported to generate ROS [189], induce the expression of specific miRNAs and reduce others. Differentially expressed levels of miRNAs have been detected in exosomes derived from cerebrospinal fluid (CSF) of young-onset AD patients compared to healthy subjects [190]. In particular, miR-125b-5p, which is abundantly expressed in the brain, was upregulated. It has been reported that miR-125b-5p leads to a significant overexpression and hyperphosphorylation of tau, indicating a connection between this miRNA and the progression of AD [191]. Interestingly, overexpression of miR-125b enhanced oxidative stress in mouse neuroblastoma Neuro2a APPSwe/D9 cells by decreasing the level of SOD [192]. This evidence supports a possible correlation between miR-125b and oxidative stress in neuron degeneration. However, a recent study shows that miR-125b-5p induces the downregulation of the expression of β-secretase and attenuates the Aβ-induced oxidative stress [193].

Oxidative stress is considered one of the most prevalent mechanisms driving neuronal death in Parkinson’s disease (PD), an age-related multifactorial neurodegenerative disorder mainly characterized by dopaminergic neuronal degeneration in the substantia nigra pars compacta. A PD hallmark is the presence in the brain of Lewy bodies that are prevalently formed by the abnormal accumulation and aggregation of misfolded α-synuclein. In PD dopaminergic neurons, oxidative stress is derived from various sources such as mitochondrial dysfunction, dopamine metabolism, impairment of the antioxidant defense mechanisms and α-synuclein aggregation [194]. Recent evidence indicates a key role of EVs, specifically exosomes, in the pathogenesis of PD. Dopaminergic neurons-derived exosomes can deliver toxic misfolded α-synuclein to healthy cells, thus spreading pathological alterations [195]. Furthermore, α-synuclein aggregates contribute to exacerbate oxidative stress, which, in turn, induce α-synuclein aggregation, thus promoting PD progression [194]. Therefore, although the release of α-synuclein by exosomes could represent a protective mechanism allowing cells to remove protein accumulation, the transfer via exosome of α-synuclein to other cells could contribute to the spreading of pathologic molecules inducing oxidative stress. Exosomes are also implicated in the PD development due to their role as miRNA carriers. Numerous miRNAs have been reported to target genes directly or indirectly linked to PD [187]. Many of these miRNAs can contribute to increase PD-associated oxidative stress by different mechanisms. For instance, some miRNAs have been implicated in promoting α-synuclein accumulation, other miRNAs downregulate genes coding for regulators of the mitochondria quality control system and others downregulate Nrf2 expression, potentially contributing to an impaired antioxidant response [187]. Interestingly, the level of several miRNAs has been found altered in circulating exosomes in PD patients. Gui et al. evaluated the miRNA profiles in CSF exosomes of PD subjects and found that 16 of them were upregulated and 11 were downregulated, when compared with CSF from healthy subjects [196]. A recent study demonstrated that miR-137 is upregulated in serum-derived exosomes of PD mice and this miRNA directly targets and negatively regulates the expression of oxidation resistance-1 (OXR1) gene in PD neurons, inducing oxidative stress [197]. Coherently, blocking exosomal miR-137 with miR-137 antagomir alleviates oxidative stress damage by up-regulating OXR1. Taken together, these data reveal that exosomes are strongly involved in the pathogenesis of PD through several mechanisms, including propagation of toxic proteins across the brain and the release of miRNAs between cells, both leading to oxidative stress.

Oxidative stress plays a complex role in tumorigenesis and cancer development [198]. The alteration of the redox balance can induce carcinogenesis and the high rate of proliferation of cancer cells induces a higher production of ROS which, in turn, increases tumor cell proliferation. However, tumor cells produce high level of antioxidants to prevent the production of ROS, which can trigger apoptosis or senescence. In addition, high levels of antioxidants can lead to resistance to cancer therapy [198]. The redox status of cancer cells is fundamental not only for intracellular processes, but also extracellularly to maintain a suitable tumor microenvironment necessary for tumor growth and invasiveness [198]. Many studies support a crucial role of EVs in modulating the redox tumor environment in order to create and sustain the hypoxic tumor microenvironment [199,200,201]. Consistent with this, much evidence indicates that hypoxia-derived tumor exosomes contribute to tumor growth, progression and are associated with increased tumor aggressiveness and metastasis [202,203]. The cargo of tumor-derived exosomes released under hypoxic conditions include active proteins such as transcription factors which promote the expression of genes associated with tumor growth and progression, RNA transcripts of redox proteins and miRNAs. The uptake of these molecules alters the redox status of target cells transferring tumorigenic properties to the healthy recipient cells [198]. In addition to mediate local signaling within the primary tumor microenvironment, EVs, and particularly exosomes, have also been strongly implicated in long-range tumor signaling to promote the formation of a hospitable pre-metastatic niche that may foster the growth of disseminated tumor cells, thus favoring metastasis [199]. Many studies also suggest a possible role of EVs in particular exosomes, in spreading drug resistance by transferring bioactive molecules such as proteins and coding and noncoding RNAs, which have been reported to be key mediators of chemoresistance [203]. In this context, it should be considered that a high percentage of drugs used to treat cancer generate ROS and RNS, which contributes to drug cytotoxic effects. As mentioned above, doxorubicin promotes the release of EVs and these EVs can serve to remove protein aggregates. Therefore, the doxorubicin-mediated release of EVs can have opposite effects: on the one hand, it can alleviate drug-induced cytotoxicity, thus reducing the chemotherapy efficacy, conversely, it can trigger an oxidative stress response on recipient cells with detrimental consequences on healthy tissues [37]. In line with this evidence, Patel and co-workers reveal an exosome-mediated mechanism of acquired chemoresistance in pancreatic cancer (PC) cells [153]. Authors demonstrated that exosomes isolated from the media of gemcitabine-treated PC cells were able to elicit chemoresistance by both increasing the levels of SOD2 and catalase and by the miR-155-mediated downregulation of deoxycytidine kinase, a gemcitabine-metabolizing enzyme.

In addition to chemoresistance, EVs have also been implicated in radioresistance. Radiation therapy is one of the main therapeutic treatments for cancer. The cytotoxicity of ionizing radiation (IR) has been attributed mainly to the production of ROS, which then cause DNA damage and organelles dysfunction [204]. Radioresistance represents a recurrent problem after the treatment [205]. Evidence clearly indicates that IR-treated cells release exosomes that affect the functions of neighboring non-irradiated cells, disseminating IR-induced effects, such as ROS increase [206]. Jabbari et al. reported that X-ray irradiated MCF-7 cells show a dose-dependent increase in ROS production, exosome biogenesis and secretion compared to control cells. They speculated about a possible ROS-mediated cellular response to therapy due to exosome increased release from irradiated cells, that can transmit resistance against radiotherapy [205].

Although the involvement of EVs in the pathophysiology of oxidative stress-related diseases has clearly emerged and many molecules responsible for this correlation, including proteins, transcription factors and noncoding RNAs, have been identified, the molecular mechanisms underlying the complex role of EVs in the development and progression of these diseases have yet to be fully elucidated.

## 5. Conclusions

In this review, we report evidence showing that EVs play an important role in the cellular response to oxidative stress. The release and the cargo of EVs are markedly affected by redox status of the originating cell. However, no universal biochemical features of EVs released under oxidative stress has been identified, as EV molecular profiles depend on different oxidative stress conditions, on the cell type and on subclass of EVs analyzed. Nevertheless, most of the studies reported in this review reveal that EVs released under oxidative stress conditions are enriched in oxidatively damaged molecules and in transcription factors, RNAs and enzymes regulating the cellular response to oxidative stress. The oxidative stress-related molecular cargo of EVs can be transferred to neighboring or distant cells, thus modulating proliferation and survival, energy metabolism and the response to oxidative stress. The evidence from in-vitro studies is in line with biochemical analyses of circulating EVs from patients with oxidative stress-related diseases, showing that in these pathological situations circulating EVs are carriers of oxidized molecules and/or molecules involved in redox metabolism. The detailed knowledge of the biochemical and biological properties of EVs released under oxidative stress is an important target for therapeutic and diagnostic purposes. The pharmacological induction of EV release might alleviate cellular damage caused by an overloading of oxidized harmful molecules. Conversely, it has been taken into account that the impact that oxidative stress-related EVs could have on target cells that is strictly dependent on EV cargo. Consistently, the detailed information regarding the specific cargo of oxidative stress-related EVs can permit us to predict their effects on target cells and could be useful as diagnostic tools, as oxidative stress is a common molecular mechanism underlying several pathological conditions.

## Data Availability

Not applicable.
